# Structural Basis of Oligomerization of N-Terminal Domain of Spider Aciniform Silk Protein

**DOI:** 10.3390/ijms21124466

**Published:** 2020-06-23

**Authors:** Rusha Chakraborty, Jing-song Fan, Chong Cheong Lai, Palur Venkata Raghuvamsi, Pin Xuan Chee, Ganesh Srinivasan Anand, Daiwen Yang

**Affiliations:** Department of Biological Sciences, National University of Singapore, 14 Science Drive 4, Singapore 117543, Singapore; rusha1989@gmail.com (R.C.); dbsfjs@nus.edu.sg (J.-s.F.); astrolai@gmail.com (C.C.L.); raghuvamsi@u.nus.edu (P.V.R.); dbscpx@nus.edu.sg (P.X.C.); dbsgsa@nus.edu.sg (G.S.A.)

**Keywords:** spider silk protein, protein self-assembly, protein structure, silk formation, protein oligomerization, NMR spider spectroscopy

## Abstract

Spider silk is self-assembled from water-soluble silk proteins through changes in the environment, including pH, salt concentrations, and shear force. The N-terminal domains of major and minor ampullate silk proteins have been found to play an important role in the assembly process through salt- and pH-dependent dimerization. Here, we identified the sequences of the N-terminal domains of aciniform silk protein (AcSpN) and major ampullate silk protein (MaSpN) from *Nephila antipodiana* (*NA*). Different from MaSpN, our biophysical characterization indicated that AcSpN assembles to form large oligomers, instead of a dimer, upon condition changes from neutral to acidic pH and/or from a high to low salt concentration. Our structural studies, by nuclear magnetic resonance spectroscopy and homology modelling, revealed that AcSpN and MaSpN monomers adopt similar overall structures, but have very different charge distributions contributing to the differential self-association features. The intermolecular interaction interfaces for AcSp oligomers were identified using hydrogen–deuterium exchange mass spectrometry and mutagenesis. On the basis of the monomeric structure and identified interfaces, the oligomeric structures of AcSpN were modelled. The structural information obtained will facilitate an understanding of silk fiber formation mechanisms for aciniform silk protein.

## 1. Introduction

Silk production from the opisthosoma glands is the most diagnostic apomorphy of the spider. The spider uses its silk for almost all essential purposes of life, such as web building, locomotion, prey capture, egg protection, etc. Owing to its outstanding mechanical properties, lightness, biocompatibility, and biodegradability, spider silk has been identified as a potent biomaterial [1,2,3,4]. As the farming of spiders and, thus, the collection of silk from natural sources is not possible, due to spiders’ cannibalistic natures [5], the artificial production of spider silk is mandatory for industrial applications. To make spider silk available on a large scale, various recombinant methods have been attempted, but with limited success, due to a poor understanding of the molecular mechanisms of silk fiber formation and silk structure–property relationships [6,7,8,9,10].

Spider silk is an orderly assembly of one or more types of spider silk proteins or spidroins. Spidroins are large protein molecules (250–350 kDa) made up of multiple repetitive (RP) domains between two regulatory terminal domains [11,12,13,14,15,16]. The RP domains account for ~90% of a silk protein in molecular weight and is responsible for the strength and elasticity of the silk [14,15]. The C-terminal domain (CTD) is relatively conserved among different types of silk proteins [17] and plays an important role in maintaining a spidroin in a solution-competent form by dimerization [18,19,20]. The CTD also reduces unspecific aggregation of a spidroin during its storage and ensures the correct alignment of the RP domains to form a well-defined fiber assembly during fiber formation [18,20].

Besides the CTD, the N-terminal domain (NTD) also plays a pivotal role in preventing the premature aggregation of a spidroin [21] that is stored in the sac region of the spider’s silk gland at a very high concentration (up to ~50% *w*/*w*) [22]. From the sac to the spinneret, the spidroin undergoes several environmental changes, such as the decrease in pH, drop in ionic strength, and reduction in spinning duct diameter [1]. These alterations change the assembly patterns of the spidroin and triggers fiber formation. In vitro studies showed that, at high pH and ionic strength, a condition corresponding to that of the lumen of a silk gland, the NTD of major ampullate spidroin (MaSpN) exists as a monomeric form [23,24]. As pH and salt concentration decrease from the lumen to the exit of the spinneret, MaSpN is converted from a monomeric form to an anti-parallel dimeric form [21,23,25,26,27]. The MaSpN structure of *E. australis* (*EA*) shows an uneven distribution of charged residues on the protein surface [21]. The positively and the negatively charged residues are clustered on the two ends of the helix bundle, creating a dipole. The salt- and pH-dependent dimerization of MaSpN indicates that, at pH 6.3, a complementary surface area is exposed to favor dimerization [23], which is not available at higher pH. Titratable residues on the protein surface play a key role in tightly controlling the dimerization process [28]. It has been proposed that MaSpN dimerization facilitates the interactions and alignments of major ampullate spidroin molecules (full-length protein) by forming long multimeric strands [25,29], and the application of shear stress on the collection of the multimeric strands initiates fiber formation.

The protonation of carboxylic residues has been suggested to be important for the dimerization of MaSpN from *EA* and *Latrodectus Hesperus* (*LH*) [26,28]. The NTD of minor ampullate spidroin (MiSpN) has also been shown to follow the same mechanism as proposed for the MaSpN, due to the conservation of most charged residues between the two types of NTDs [30]. However, the NTDs from other types of silk proteins, such as aciniform spidroin (AcSp) and flagelliform spidroin (FlSp), have less than 40% sequence identity with MaSpN and MiSpN [31]. The numbers of charged residues, which play crucial roles in MaSpN and MiSpN dimerization, are significantly smaller than those in AcSpN. Therefore, the assembly pattern and properties of AcSpN could be completely different from those of MaSpN. Until now, however, no studies on the structure of AcSpN and its self-assembly mechanism have been reported.

Here, we identified the NTD sequences of aciniform and major ampullate spidroins from the golden orb weaving spider, *Nephila antipodiana* (*NA*). Using NMR spectroscopy, we determined the three-dimensional (3D) structure of AcSpN in the presence of 500 mM NaCl at neutral pH. Using biophysical techniques, we found that AcSpN and MaSpN from *NA* have very different oligomerization properties at low salt concentrations. Using mutagenesis and hydrogen–deuterium exchange mass spectrometry, we identified the oligomerization interfaces and obtained structural models of AcSpN dimer and larger oligomers.

## 2. Results and Discussion

### 2.1. Sequences of AcSpN and MaSpN from NA

**Figure 1:**
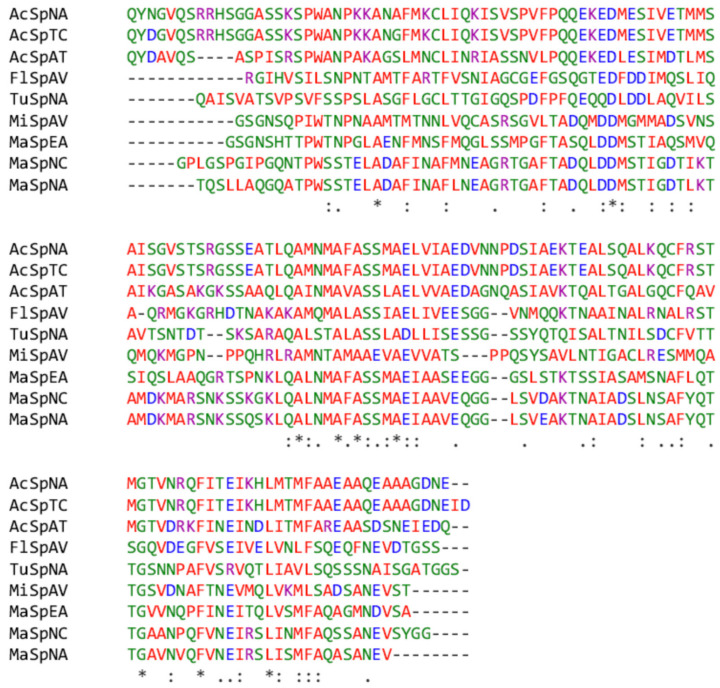
Sequence alignment of N-terminal domains of different types of spider silk proteins, including aciniform: AcSpNA, AcSpTC (genbank: AWK58691.1), and AcSpAT (AHK09776.1); major ampullate: MaSpNA, MaSpNC (PDB: 5IR2), and MaSpEA (PDB: 3LR2); minor ampullate: MiSpAV (PDB: 2MX8); flagelliform: FlSpAV (GBM14626.1); tupliform: TuSpNA (ACI23395.1). “*”, “:”, and “.” indicate perfect alignment, strong similarity and weak similarity, respectively.

Based on previous our knowledge of RP and NTD sequences, the AcSpN and MaSpN sequences from *NA* were identified by PCR and RACE-PCR, as shown in Figure 1. According to pairwise sequence alignment, AcSpN from *NA* shares 95% and 58% amino acid sequence identities with AcSpNs from *Trichonephila clavipes* (*TC*) and *Argiope trifasciata* (*AT*), respectively, but it shares only 34–37% sequence identities with the MaSpNs from *NA*, *EA,* and *N. clavipes* (*NC*). MaSpN from *NA* shares 88% and 90% identities with the MaSpNs from *EA* and *NC*, respectively, as shown in Figure 1. The results show that NTDs share high sequence identities within the same type of silk proteins from different spider species, but low identities between different types of silk proteins within the same species. Based on sequence comparison with the MaSpNs from *EA* and *NC*, the structural regions of AcSpN (S15-E153) and MaSpN (T1-E137) from *NA* were predicted and further used for cloning, protein production, and biophysical characterization. The AcSpN construct (S15-E153), used for the biophysical characterization, is hereafter renumbered as S4-E142.

### 2.2. Effects of Salt Concentration and pH on Oligomerization of MaSpN and AcSpN

**Figure 2:**
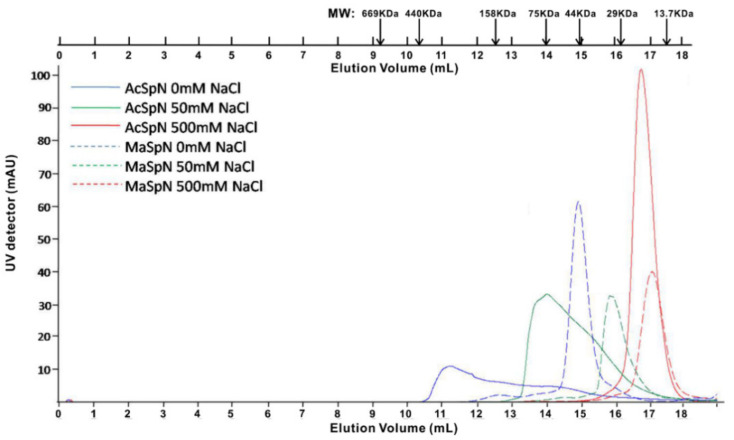
Size exclusion chromatograms of AcSpN and MaSpN at pH 7.0 and three different NaCl concentrations. The molecular weight markers of standards are placed on the top of the experimental profiles.

After thrombin cleavage, purified MaSpN and AcSpN contained 140 and 142 residues, respectively due to additional three residues (GSM) (at the N-terminus) coming from the cloning vector. The molecular weights (MW) of MaSpN and AcSpN were 14.7 and 15.3 kDa, respectively. According to our size exclusion chromatography (SEC) experiments, the MaSpN from *NA* at pH 7.0 and 500 mM NaCl eluted at a volume consistent with that for a monomeric form (~15 kDa), as shown in Figure 2. As the salt concentration was reduced to 50 mM, the elution volume decreased to a value corresponding to a dimeric form (~30 kDa). This transformation from a monomeric to dimeric form, due to the decrease in salt concentration, is in accordance with previously studied MaSpN and MiSpN from other spider species [23,24,28]. Lowering the salt concentration in the gel filtration buffer to zero resulted in a further decrease in the elution volume, corresponding to ~44 kDa. From this decrease, we cannot conclude that the protein undergoes further oligomerization because the elution volume decrease might be caused simply by non-specific protein–matrix interactions in the absence of salt [32].

AcSpN from *NA* at 500 mM NaCl eluted as a single sharp peak, as shown in Figure 2. In the presence of 50 mM NaCl, the elution profile of AcSpN was very different from that of MaSpN. Instead of a single sharp peak, the protein eluted as a broad asymmetric peak, corresponding to a MW range of 20–100 kDa. The further decrease in salt concentration to 0 mM made the elution peak even broader, corresponding to a MW range of 30–400 kDa. The results suggest that AcSpN forms a mixture of different oligomers at low NaCl concentrations (≤50 mM).

**Figure 3:**
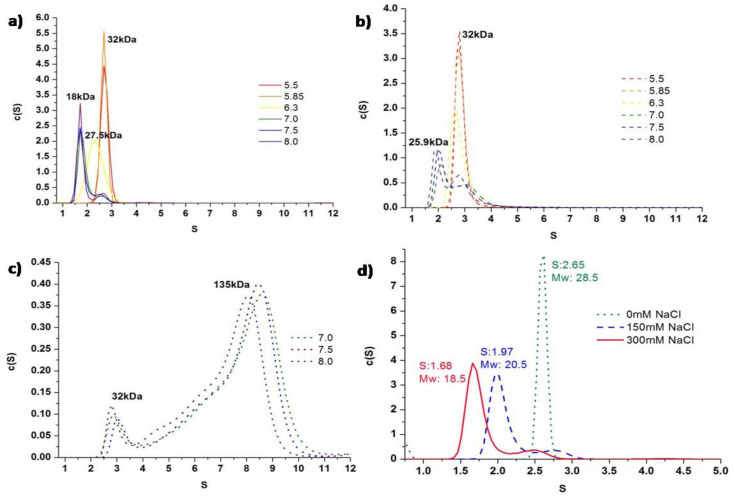
Sedimentation profiles of AcSpN and MaSpN. Overlay of sedimentation profiles of AcSpN at varying pH in the presence of 300 mM NaCl (**a**), 150 mM NaCl (**b**), and 0 mM NaCl (**c**). Overlay of sedimentation profiles of MaSpN at pH 7.0 and in the presence of varying NaCl concentrations (**d**).

To determine the number of molecules forming the oligomers, analytical ultracentrifugation (AUC) experiments were performed. From the experiments, at a pH range of 5.5 to 8.0 and NaCl concentrations of 0, 150, and 300 mM, distributions of the sedimentation coefficient (S) were obtained, as shown in Figure 3. The S-value of the dominant AcSpN peak was 1.7 at 300 mM NaCl and in a range of pH 7.0–8.0, which corresponds to a MW of ~18.0 kDa, as shown in Figure 3a. This value is very close to the monomer size of AcSpN (15.3 kDa). As the pH was reduced to 5.85 and 5.50, the S-value increased to 2.8, corresponding to a MW of ~32 kDa, or the size of AcSpN dimer, when the ionic strength remained unchanged. At pH 6.3, a broader peak at an S value of 2.3 was recorded. This peak corresponds to neither a monomeric nor a dimeric form, implying dynamic equilibrium between the monomeric and dimeric forms, or fast exchange between the two forms.

At 150 mM NaCl, AcSpN existed in a dimeric form at pH < 7.0, while it existed as a mixture of dimeric and monomeric forms that may undergo exchanges at pH ≥ 7.0, as shown in Figure 3b. In the absence of NaCl, sedimentation data at pH < 7.0 could not be recorded because of protein precipitation. Between pH 7.0 and 8.0, AcSpN sedimentation profiles displayed two obvious peaks, one corresponding to a MW of ~32 kDa (dimer) and the other to ~135 kDa (nine-mer), as shown in Figure 3c. In addition, there was a very broad peak, with S-values from 3.5 to 7 in each of the profiles, demonstrating the presence of multiple oligomers with different molecular weights and dynamic equilibria among the oligomers, consistent with the gel filtration result. In the absence of NaCl, backbone amide ^1^H-^15^N correlation signals of AcSpN were not detectable in the 2D NMR HSQC spectrum, further showing that AcSpN exists mainly in large oligomers in a 20 mM phosphate buffer without NaCl at pH 7.0.

The sedimentation profiles obtained from purified MaSpN at pH 7.0 and varying salt concentrations were also evaluated, as shown in Figure 3d. In the absence of NaCl, a sharp single peak at an S-value of 2.65 was recorded, corresponding to a MW of ~28.5 kDa (dimeric form). As NaCl concentration increased to 150 mM, the S-value decreased to 1.95 (corresponding MW: ~20.5 kDa). The further increase in NaCl concentration to 300 mM reduced the S-value to 1.68 (corresponding MW: 18.5 kDa) and resulted in a monomeric form of MaSpN. The AUC experiments showed that, unlike AcSpN, the MaSpN did not form any high order assembly larger than dimers. This conclusion agrees with that drawn from the studies on MiSpN from *AV* [30] and the MaSps from *EA*, *NC, LH,* and *Latrodectus mactans* [23,26,27].

### 2.3. Structures of AcSpN and MaSpN Monomers from NA

Our AUC and size-exclusion chromatography data show that AcSpN forms a stable monomer at neutral pH and high ionic strength. So, a ^13^C,^15^N-labeled AcSpN (142 residues, including three residues from the vector) sample, with a 20 mM phosphate buffer (pH 7.0) and 500 mM NaCl, was used to determine the 3D structure of the AcSpN monomer by NMR spectroscopy. Based on 2D ^1^H-^15^N HSQC, 3D HNCACB, and CBCA(CO)NH, 128 out of 142 residues were assigned in backbone resonances (excluding ^13^CO). The first five residues, including three from the vector (G1, S2, M3, S4, and S5), and four residues from the unstructured region (T56, S57, S60, and Q100), could not be assigned. The ^15^N chemical shifts of five prolines were not assigned. Using 3D MQ-(H)CCH-TOCSY, H(CCO)NH, 3D CC(CO)NH, and NOESY spectra, most aliphatic and some aromatic sidechain spins were assigned. Excluding ^13^CO, the overall completeness in chemical shift assignments was ~83%. The NMR resonance assignments were deposited in the Biological Magnetic Resonance Bank (BMRB, ID: 36341). In total, 1598 distance restraints derived from NOE assignments and 204 backbone dihedral angle restraints, generated from TALOS analysis, were used to calculate the structure of AcSpN. The 20 lowest energy structures had a backbone RMSD value of 0.94 ± 0.23 Å. A Ramachandran plot also showed that 99.6% of the calculated backbone dihedral angles were in the allowed region. The experimental restraints are summarized in Table S1 and the 20 lowest energy structures were deposited in the Protein Data Bank (PDB ID: 7BUT).

**Figure 4:**
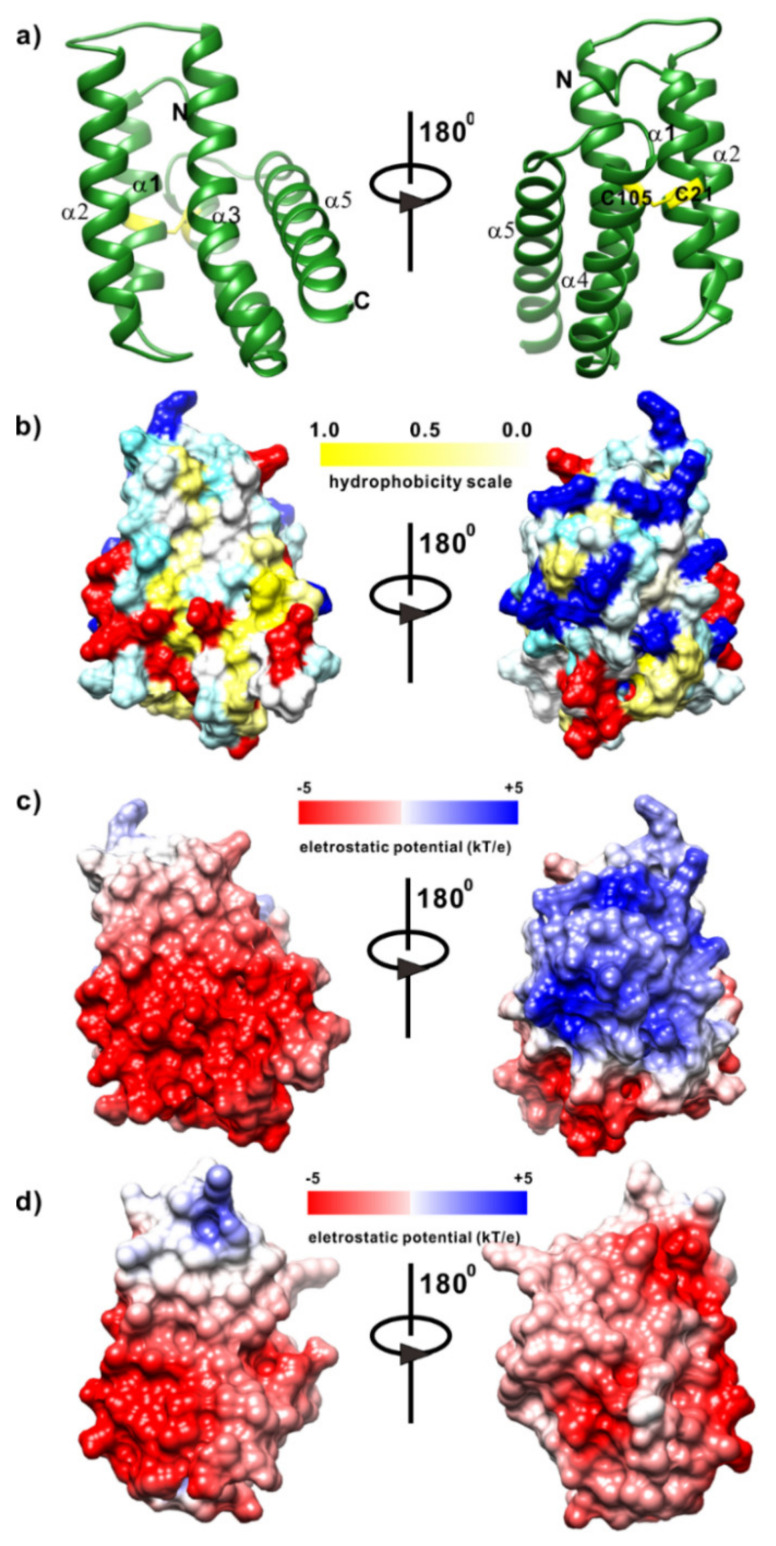
Ribbon (**a**), hydrophobicity (**b**), and surface charge potential (**c**) representations of AcSpN monomer, and surface charge potential representation of MaSpN from *NA* (**d**). Disulfide between C21 and C105 is indicated by yellow sticks in (**a**). Red and blue colors represent negatively and positively charged residues, respectively, in (**b**).

AcSpN adopts a helical bundle fold with five helices: α1 (14–27), α2 (34–54), α3 (62–81), α4 (88–108) and α5 (115–138), as shown in Figure 4a. The first 13 and last 4 residues were disordered. The only tryptophan residue at the ninth position (W9) is located inside the cavity created by the five helices. Unlike MaSpNs, AcSpN contains two cysteine residues, C21 located at helix 1 and C105 located at helix 4, which are not conserved, as shown in Figure 1. These two residues form an intra-molecular disulfide bond which holds the two helices together, as shown in Figure 4a. Most hydrophobic residues are packed among helices, suggesting hydrophobic interactions are the dominant force to hold helices together tightly. Only a few hydrophobic residues located on helix 3 are surface exposed, as shown in Figure 4b.

MaSpNs from *NA* and *NC* share 88% sequence identity. Due to such a high identity, we modelled the structure of MaSpN from *NA* at the Swiss-model Server [33], using the structure of MaSpN from *NC* (PDB: 5IZ2) as a template. The modelled structure is shown in Figure S1a. Similar to AcSpN, hydrophobic interactions are the dominant force to hold helices tightly, and only a few hydrophobic residues located on helix 3 are surface exposed, as shown in Figure S1b.

Despite their low sequence similarity, AcSpN and MaSpN are very similar in overall structure, as shown in Figure 4a and Figure S1a. The main difference lies in surface charge distribution, as shown in Figure 4c,d. AcSpN contains significantly more charged residues (12 Arg + Lys and 17 Asp + Glu) than MaSpN from *NA* (8 Arg + Lys and 14 Asp + Glu), as shown in Figure 1. For AcSpN, the negatively charged residues are located mainly on the surface formed by the α2, α3, and C-terminal half of α5, as shown in the left panels of Figure 4a,c, while the positively charged residues are distributed mainly on the opposite side, formed by α1 and α4, as shown in the right panels of Figure 4a,c. In addition, the N-terminal end is positively charged, while the C-terminal end region is negatively charged, as shown in Figure 4c. For MaSpN, except for one side of the N-terminal end region, all other parts of the surface are negatively charged, as shown in Figure 4d. This charge distribution of MaSpN from *NA* is similar to that of MaSp from *EA* [21].

Because the dimerization of MaSpN and oligomerization of AcSpN are affected by salt concentration, as shown in Figure 2 and Figure 3, the dominant force to mediate oligomerization must be electrostatic interactions. After two MaSpN molecules come together to form a dimer in an antiparallel way through electrostatic interactions [21], the surface of the dimer is nearly fully negatively charged, as shown in Figure S2. Such a dimer should be stable and cannot further assemble to form larger oligomers, due to electrostatic repulsion between the dimers. Different from MaSpN, no matter how two AcSpN molecules are arranged to form a dimer, the surface of the dimer still contains both negatively and positively charged patches, provided that all the acidic and basic residues are assumed to be fully charged. Consequently, the AcSpN dimer can further assemble to form larger oligomers under lower salt concentrations.

### 2.4. Identification of Oligomerization Interfaces for AcSpN

**Figure 5:**
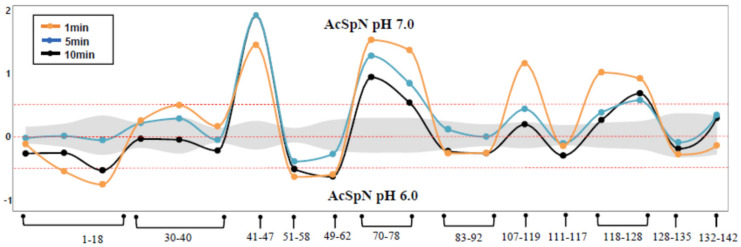
Differences in average deuterons exchanged (*Y*-axis) in AcSpN at pH 7.0 and pH 6.0 for three different exchange times. Each pepsin proteolyzed peptide is listed from N to C terminus (*X*-axis). Peptides spanning continuous regions are grouped by brace brackets. Empirically determined 0.5 Da difference in deuterium uptake is considered as significant (dashed lines). Positive and negative differences in deuterium exchange represent increased and decreased exchange, respectively, in the AcSpN from pH 6.0 to pH 7.0 by the respective peptides. Standard deviations are shaded gray. Orange, blue, and black dots and lines represent exposure times of 1, 5, and 10 min at pH 7.0 and 10, 50, and 100 min at pH 6.0.

To identify oligomerization interface, we used hydrogen–deuterium exchange (HDX) mass spectrometry (MS). HDXMS is a powerful technique to probe the conformational dynamics of proteins as a function of deuterium uptake by protein backbone amide hydrogen, and the deuterium uptake depends on hydrogen bonding propensity and solvent accessibility [34]. HDXMS has been employed to identify the binding hotspots on proteins in various contexts, such as protein–protein, protein–lipid, and protein–nucleic acid interactions, etc. [35]. As shown by AUC, AcSpN forms predominantly dimers at pH 6.0 compared to pH 7.0 at a constant salt concentration of 150 mM NaCl, as shown in Figure 3b, which were the sample conditions used in our HDX experiments to map the binding interface. As pH influences the deuterium uptake kinetics (i.e., the HDX rate is reduced by one order of magnitude when the pH is decreased from 7.0 to 6.0) [36], we extended the deuteration time by a factor of 10 for direct comparison between the pH 7.0 and 6.0 samples. In total, 73.9% sequence coverage of pepsin proteolysed peptides of AcSpN was achieved in our HDXMS experiments in both of the pH states, as shown in Figure S3. The differences of deuterons taken by the protein at pH 7.0 and 6.0 are shown in Figure 5 for three different incubation times. Fragments 41–47 are located in α2, 70–78 in α3, 107–110 in α4, and 118–128 in α5 incorporated significantly more deuterons at pH 7.0 than at pH 6.0 for the early time point of one minute. For longer incubation times (5 and 10 min), only fragments 41–47, 70–78, and 118–128 remained to have more deuterons at pH 7.0 than at pH 6.0.

According to circular dichroism data of AcSpN, as shown in Figure S4, AcSpN adopts identical secondary structures in the presence of 150 mM NaCl at pH 6.0 and 7.0. On the basis of our AUC data, as shown in Figure 3b, the dimer and monomer populations are different at pH 6.0 and 7.0. So, the difference in the deuterium incorporation level reflects that different AcSpN fragments have different solvent accessibility in the monomeric and dimeric forms. The four regions showing the reduced level of deuterium exchange by lowering pH may all be involved in the dimerization interface, but the three regions (41–47, 70–78, and 118–128) in α2, α3, and α5 are more likely, since they had lower deuterium levels at pH 6.0 than at pH 7.0 for all three incubation times.

### 2.5. Structural Models of AcSpN Dimers and Oligomers

**Figure 6:**
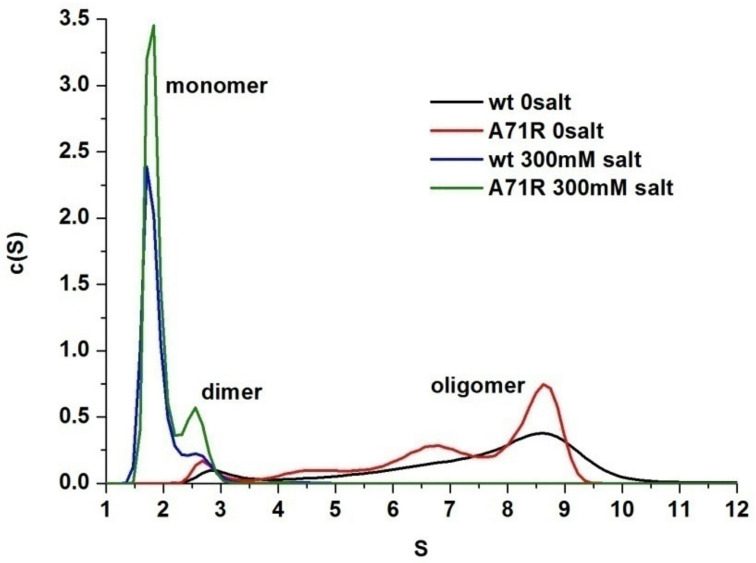
Overlay of sedimentation profiles of AcSpN and its A71R mutant at pH 7 and in the presence and absence of NaCl.

Assuming that 41–47, 70–78, and 118–128 are located in the dimer interface of AcSpN, we modelled the dimeric structure using HADDOCK [37]. The active residues used in the calculation are listed in Table S2. The arrangements of the two AcSpN units in this modelled structure, as shown in Figure S5, are similar to those of the two MaSpN from *EA* in the dimeric structure, solved by X-ray crystallography [21]. A previous study on MaSpN from *EA* has demonstrated that its A72R mutant exists as a monomeric form in a pH- and salt-independent way [24]. A72 is conserved among MaSp, MiSp, AcSp, TuSp, and FlSp from different species, as shown in Figure 1, which corresponds to the A71 of AcSp from *NA*. Similar to MaSpN, the sidechains of A71 and A71′ are nearby in the modelled dimer structure, as shown in Figure S5. If the modelled structure were correct, the A71R mutant of AcSpN would exist mainly in a monomeric form because R71 and R71′ repel each other to dissociate the two units. To examine whether the modelled structure is correct or not, we prepared an A71R mutant. The sedimentation profiles of the A71R mutant were very similar to those of WT, as shown in Figure 6, and the mutation did not prevent the oligomerization of AcSpN at pH 7.0. Even in the presence of 300 mM NaCl, ~10% of the A71R mutant still existed in a dimeric form, similar to the WT protein. Clearly, the effect of this mutation on AcSpN was completely different from that on MaSpN. The experimental result demonstrates that the modelled dimeric structure, shown in Figure S5, is not the dominant conformation at neutral pH.

**Figure 7:**
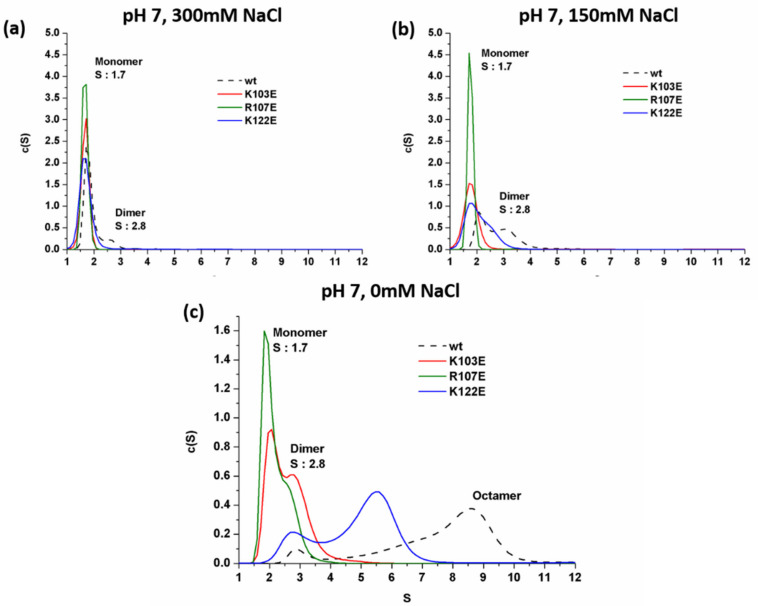
Overlay of sedimentation profiles of WT AcSpN, K103E, R107E, and K122E mutants at pH 7.0 in the presence of 300 mM (**a**), 150 mM (**b**), and 0 mM (**c**) NaCl.

To test if fragment 107–110 in α4 is also involved in the dimeric interface, we mutated a positively charged R107 in α4 into a negatively charged glutamic acid residue, which is exposed on the surface of the monomeric structure and located in the positively charged region. In addition, K103 was also chosen for mutation because it is exposed and located in α4, but it was not covered by the HDXMS, as shown in Figure S3. As a comparison, K122 in α5 was mutated too, since it is partially solvent-exposed and not in the interface of the dimer described above, as shown in Figure S5. In the presence of 300 mM NaCl, K103E, R107E, and K122E were predominantly in a monomeric form at pH 7.0, as shown in Figure 7a, similar to the WT AcSpN. Under 150 mM NaCl, WT and K122E existed as a mixed population of dimer and monomer, whereas the other two mutants were still predominantly in a monomeric form, as shown in Figure 7b. Further complete removal of NaCl could not covert K103E and R107E into oligomers larger than dimers. Instead it shifted to a mixed population of dimers and monomers, as shown in Figure 7c. However, the mutation effect on K122E was not so significant. After the removal of NaCl, K122E could form oligomers larger than a dimer, although it formed oligomers smaller than WT AcSpN. Taking the results from the mutation and HDXMS together, the positively charged region of α4 is also involved in the dimeric and oligomeric interface.

**Figure 8:**
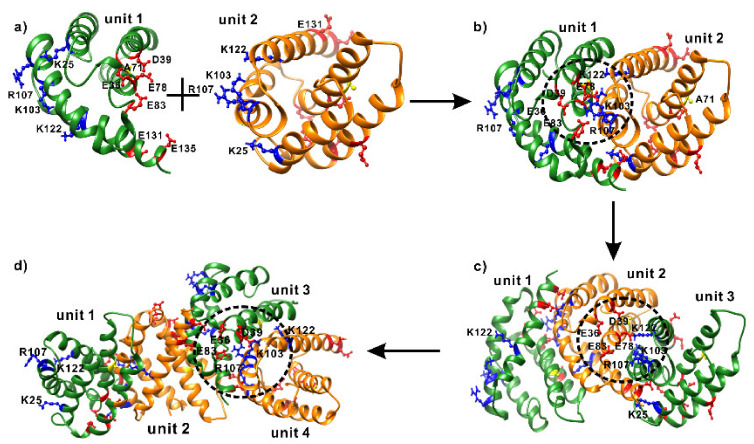
Oligomerization of AcSpN from monomer (**a**) to dimer (**b**), trimer (**c**), and tetramer (**d**). Positively and negatively charged residues located in the interfaces are displayed in blue and red sticks-and-balls, respectively. In addition, A71 is highlighted in yellow sticks-and-balls. K122, which was selected for mutation but is not in direct intermolecular charge–charge interactions, is also highlighted.

Assuming that 41–47, 70–78, 102–110, and 118–128 are in the interface, we modelled the dimeric structure again using HADDOCK and obtained a totally different structural model, as shown in Figure 8a. In this structure, α2, α3, and α5 in unit 1 interact with α1 and α4 in unit 2 through electrostatic attractions, including E36^1^(α1)-R107^2^(α4), D39^1^(α1)-R107^2^(α4), E83^1^(α3-α4 loop)-R107^2^(α4), E78^1^(α3)-K103^2^(α4), E131^1^(α5)-K25^2^(α1), and E135^1^(α5)-K25^2^(α1), where superscripts 1 and 2 represent units 1 and 2, respectively. As a result, fragments 41–47, 70–78, 92–108, and 118–128 are more buried from solvent in the dimeric than monomeric structure, as shown in Figure 8a,b, suggesting that 41–47, 70–78, 102–110, and 118–128 have slower HDX rates or lower deuterium levels in the dimeric form than in the monomeric form. This is consistent with the HDXMS data, as shown in Figure 5. The modelled structure also agrees with the mutation results, as K103 and R107 are located in the dimeric interface and contribute to dimer stability through interactions with negatively charged residues, while K122 in unit 2 is far away from the negatively charged residues in unit 1 and does not make a significant contribution to the dimer stability. In this model, A71^1^ in unit 1 does not face A71^2^ in unit 2, explaining why A71R has a similar oligomerization property to WT AcSpN. Therefore, the modelled structure, shown in Figure 8b, is the dominant dimeric conformation of AcSpN.

The dimeric structure, shown in Figure 8b, carries both positive and negative charges on its surface. Using the same dimerization mode between unit 1 and unit 2, unit 3 can interact with unit 2 in the dimer to form a trimer, as shown in Figure 8c, and then to form a tetramer, as shown in Figure 8d, through interactions of unit 3 in the trimer with unit 4. In this way, AcSpN can assemble to form larger oligomers. It is noteworthy that there are clashes between some sidechains located in the protein–protein interfaces for the current structural models. The clashes can be eliminated by performing molecular dynamics simulations. In terms of enthalpy, the larger the oligomer, the more stable the oligomer. In terms of entropy, however, the larger the oligomer, the less stable the oligomer. Considering both entropy and enthalpy, only oligomers in a certain range of molecular weights can exist. Therefore, the size distribution of the oligomers depends on the sample conditions, such as pH and salt concentration, which mainly affect the electrostatic interactions or enthalpy of an oligomer.

Together with the dimerization feature of MaSp CTD [20], the pH-dependent dimerization of the MaSp NTD has been proposed to be essential to the self-assembly of full-length MaSp into multimers, induced by lowing pH and/or salt concentration [25,29]. Unlike CTD and NTD, which are well-folded, the RP core region of MaSp is disordered, contains multiple (GA)_n_ and (A)_n_ motifs, and has relatively low water-solubility [38]. A recent study has suggested that the self-assembly of MaSp is facilitated by the RP region [38], since it tends to self-associate by hydrophobic interactions. Similar to the CTD of MaSp, the CTD of AcSp has already been shown to form a stable dimer [20]. Different from the RP of MaSp, the RP region of AcSp consists of multiple well-folded RP domains plus hydrophilic linkers between two RP domains, which are highly water-soluble and exist in a monomeric form [39]. Unlike the NTD of MaSp, the NTD of AcSp assembles to form large oligomers instead of dimers upon the reduction in salt concentration and/or pH. Therefore, the self-assembly or polymerization of full-length AcSp, induced by increasing protein concentration and lowering pH and salt concentration, may be solely determined by its NTD.

## 3. Materials and Methods

### 3.1. Sequence Identification, Cloning, Expression, and Purification

First, partial sequences of AcSpN and MaSpN were obtained from the genomic DNA of spider *NA* by PCR using degenerate primer sets designed on the basis of the conserved amino acid sequences of NTDs and RPs from known AcSp and MaSp genes, respectively [12,14,15,39,40]. Second, the N-terminal region sequences of AcSpN and MaSpN were identified from total RNA of spider *NA* by RACE-PCR, using RACE specific primers and gene-specific primers based on the partial sequences found in the first step.

The identified AcSpN and MaSpN sequences were sub-cloned to a modified pET-32a (Novagen) vector with a cleavable N-terminal His-tag. The sequences were confirmed by DNA sequencing. The confirmed plasmids were transformed into BL21 (DE3) *Escherichia coli* cells for over expression. For unlabeled protein expression, Luria–Broth medium was used, while for labelled protein expression M9 minimal medium was used. The M9 medium contained ^15^N-labelled NH_4_Cl and ^13^C-labelled glucose as the sole source of nitrogen and carbon, respectively. When the cell density reached an OD_600_ value of 0.5–0.6, IPTG was added to induce the overexpression of the protein. After induction, the culture grew overnight at 20 °C. The cells were harvested by centrifugation and lysed by sonication.

The AcSpN was harvested from an inclusion body and then dissolved in a 20 mM Tris buffer with 8 M urea at pH 8.0. The protein was purified by immobilized metal affinity chromatography using the same buffer containing 8 M urea. The elute was refolded by one step dialysis against the Tris buffer without urea. The His-tag of the refolded AcSpN was removed by bovine thrombin (Sigma-Aldrich, St. Louis, MO, USA) treatment. Finally, the cleaved protein was passed through a gel filtration column to obtain samples with ~95% purity. The AcSpN mutants were expressed and purified by following the same procedure. MaSpN was expressed using the same method, but it was purified under a native condition (without urea) from the supernatant by immobilized affinity chromatography and then by size exclusion chromatography.

### 3.2. Size Exclusion Chromatography

An analytical column (superdex 200 Increase 10/300 GL, GE) was used to study the salt induced assembly of AcSpN and MaSpN. The elution profiles for each protein in 20 mM sodium phosphate buffer (pH 7.0) were monitored in the presence of 0, 50, and 500 mM NaCl, respectively. The protein samples injected were of ~600 µM concentration and the fractions were collected every 0.5 mL. The fractions were analyzed using SDS-PAGE and size estimation was done by comparison with previously chromatographed molecular mass standards.

### 3.3. Analytical Ultracentrifugation

The sedimentation velocity experiments were recorded using Beckman Optima XL-I centrifuge, fitted with a four-hole AN-60 rotor at 40,000 RPM. Each sample contained 1.5 mg/mL protein in 20 mM phosphate buffer with additional NaCl. In total, 210 scans were recorded for each sample at 24 °C. Only once the vacuum was far below 100 microns and the temperature was steady, the sedimentation profile was recorded at 280 nm. The absorbance data were then fitted to continuous c(S) distribution of the Lamm equation using Sedfit program to calculate the corresponding s-value for each sample [41].

### 3.4. NMR Spectroscopy and Structure Calculation

All NMR experiments were recorded on a Bruker 800 MHz NMR spectrometer, equipped with a cryo-probe at 25 °C. The sample for structure determination contained ~1 mM ^15^N,^13^C-labelled protein in a buffer with 20 mM sodium phosphate (pH 7.0), 500 mM NaCl, 1 mM EDTA, 5% D_2_O, and 0.01% NaN_3_. To solve the structure of monomeric AcSpN, 2D ^1^H-^15^N HSQC, 3D HNCACB, CBCA(CO)NH, H(CCO)NH, C(CCO)NH, and MQ-(H)CCH-TOCSY, and 3D ^15^N/^13^C-edited NOESY data were recorded [42,43]. All the NMR data were processed using NMRPip and analyzed using NMRFAM-Sparky [44,45]. After backbone assignment using 2D ^1^H-^15^N HSQC, 3D HNCACB, and 3D CBCA(CO)NH spectra, the side chain assignment was done using 3D H(CCO)NH, C(CCO)NH, MQ-(H)CCH-TOCSY, and NOESY spectra by following the previously described methods [42,46,47]. Unambiguous NOE assignments were obtained from 3D ^15^N-edited NOESY and ^13^C-edited NOESY spectra.

The distance restraints were derived from assigned NOEs and the dihedral angle restraints were calculated using the online server TALOS+, using assigned chemical shifts of C_α_, C_β_, N, H_α_, and H_N_ [48]. The initial structure calculation was carried out with Xplor-NIH [49] using the conventional simulated annealing protocol from an extended conformation of AcSpN. Subsequently, the best models with the lowest total energy were selected for EEFX force-field implicit refinement [50]. A total of 100 structures were calculated using the EEFX refinement and 20 conformers with the lowest total energy were deposited. The quality of the structures was assessed with MolMol [51] and Procheck [52] software, and the figures were prepared with Chimera [53].

### 3.5. Hydrogen–Deuterium Exchange Mass Spectrometry (HDXMS)

Purified AcSpN was diluted to a concentration of 120 µM in 20 mM sodium phosphate buffer with 150 mM NaCl at pH 7.0 and pH 6.0, respectively. Deuterium exchange reactions were performed by diluting 2.5 μL protein sample in 22.5 μL deuterium buffer of the corresponding pH and salt to obtain a final concentration of 90% D_2_O. The deuteration reactions were done at 28 °C for 1, 5, and 10 min for the pH 7.0 protein samples and 10, 50 and 100 min for the pH 6.0 protein samples to correct for the deuterium exchange rates for 1 unit pH drop in reaction conditions. The exchange was quenched by adding 25 μL ice-cold buffer containing 0.1% TFA, which brought the final pH of the reaction mixture to 2.5. The quenched samples were injected into an immobilized pepsin column (2.1 mm × 30 mm, Poroszyme, ABI, Foster City, CA, USA) at a flow rate of 100 μL/min in 0.1% (*v*/*v*) formic acid in water solution by nanoUPLC sample manger. The pepsin proteolyzed peptides were trapped in the C18 ACQUITY BEH VanGuard column (2.1 mm × 5 mm, 1.7 μm resin; Waters). A gradient of 8% to 40% of acetonitrile in 0.1% (*v*/*v*) formic acid was used to elute the peptides at a flow rate of 40 μL/min and was resolved using the ACQUITY UPLC-BEH C18 reversed-phase column (1.0 mm × 100 mm, 1.7 μm resin; Waters). The detection and mass measurements of eluted peptides was done on a Synapt G2-Si HDXMS mass spectrometer (Waters, Manchester, UK) in the MS^E^ mode, a non-biased, non-selective CID method with constant calibration with Glu-Fibrinogen at a concentration of 200 fmol/μL (5μL/min) [36].

The identification of the peptides was carried out on ProteinLynx Global Server 2.4 software (Waters, Milford, MA, USA) [36]. The MS^E^ mode of the peptide identification was used to search against AcSpN sequence, followed by non-specific protease cleavage and non-modified amino acid options. Further, the precursor ion mass tolerance was fixed at 10 ppm and a false discovery rate of 4% was set for identification. To analyze the deuterium uptake of each peptide that fulfilled the conditions, Waters’ Dynamx 3.0 software was used. Peptides with high signal to noise ratios and distinct spectral mass envelopes were chosen to generate difference plots and to carry out further analysis. An average of three replicates are reported in the represented data.

### 3.6. Modelling of AcSpN Dimers

HADDOCK 2.2 [37] was employed to model the dimeric structures of AcSpN. The monomeric structure solved in this work was used as a template structure. The residues in the dimeric interface, which were identified from HDXMS and mutagenesis, were used as active residues. The residues within 6.5 Å around the active residues were selected as passive residues. All of the calculations were performed on the HADDOCK 2.2 webserver using the default parameters [37,54]. The best structures were chosen by HADDOCK score and Z-score and were analyzed with UCSF chimera [53].

## 4. Conclusions

In this study, we identified the AcSpN and MaSpN amino acid sequences from *Nephila antipodiana*, solved the monomeric structure of AcSpN, and obtained the structures of AcSpN oligomers, based on HDXMS mutagenesis data and the structures of *NA* MaSpN monomer and dimers, based on homology modelling. In comparison with MaSpN and MiSpN, AcSpN adopts a similar five-helix bundle fold, but carries significantly more positively and negatively charged residues and displays very different charge distribution on the protein surface. Consequently, AcSpN exists as a mixture of different oligomers at neutral pH and low salt conditions, whereas MaSpN exists as a dimer at the same conditions. We have found another inter-molecular interaction mode, through which AcSpN can assemble to form dimers, trimers, and larger oligomers. This interaction mode differs from that used by MaSpN, implying that AcSp and MaSp may adopt different molecular mechanisms in fiber formation.

## Supplementary Materials

Supplementary materials can be found at https://www.mdpi.com/1422-0067/21/12/4466/s1.

## Abbreviations

NMR	Nuclear magnetic resonance
HDXMS	Hydrogen–deuterium exchange mass spectrometry
CTD	C-terminal domain
NTD	N-terminal domain
RP	Repetitive domain
AcSp	Aciniform spidroin
MaSp	Major ampullate spidroin
AcSpN	NTD of AcSp
MaSpN	NTD of MaSp
AUC	Analytical ultra-centrifugation
HSQC	Heteronuclear single-quantum correlation
