# Supplementary material for: Structural Basis of Oligomerization of N-Terminal Domain of Spider Aciniform Silk Protein
Source: Int J Mol Sci. 2020 Jun 23;21(12):4466. doi: 10.3390/ijms21124466 (PMC7352312; doi:10.3390/ijms21124466)
Supplement: Supplementary file 1 [file ijms-21-04466-s001.pdf]

Supplementary Materials

Structural Basis of Oligomerization of N-terminal Domain of Spider Aciniform Silk Protein

Rusha Chakraborty, Jing-song Fan, Chong Cheong Lai, Palur Venkata Raghuvamsi, Pin Xuan Chee, Ganesh Srinivasan Anand, Daiwen Yang*

Department of Biological Sciences, National University of Singapore, 14 Science Drive 4, Singapore 117543

Table S1. Structural statistics for the final 20 conformers of AcSpN[a]

Distance restraints	
Intra-residue (*i-j* = 0)	368
Sequential (|*i-j*| = 1)	403
Medium range (2 ≤ |*i-j*| ≤ 4)	230
Long range (|*i-j*| ≥ 5)	106
Hydrogen bond	158
Total	1265
Dihedral angle restraints	
ϕ	108
ψ	108
Average rmsd to the mean structure (Å)[b]	
Backbone atoms	0.94 ± 0.23
Heavy atoms	1.42 ± 0.22
ϕ/ψ space[c]	
Most favored region (%)	94.4
Additionally allowed region (%)	4.8
Generously allowed region (%)	0.7
Disallowed region (%)	0.4
rmsd from covalent geometry	
Bonds (Å)	0.007 ± 0.000
Angles (deg.)	0.853 ± 0.008
Impropers (deg.)	0.552 ± 0.022
rmsd from experimental restraints	
NOEs (Å)	0.043 ± 0.002
Dihedral angles (deg.)	0.390 ± 0.077
eefx potential energy (kcal)[d]	-1614 ± 15.34

[a] Selected from 100 calculated conformers according to overall energy.

[b] Calculated with MOLMOL over secondary structure region (15-26, 37-53, 62-81, 90-109, 115-138).

[c] Calculated with PROCHECK-NMR.

[d] Refinement with eefx force-field.

Tabel S2. Active residues used in HADDOCK calculation

Type of dimer	unit	Active residues
I	A	E41-M47, M70-E78, I118-F128
	B	E41-M47, M70-E78, I118-F128
II	A	E41-M47, M70-E78
	B	L102-M110, I118-F128

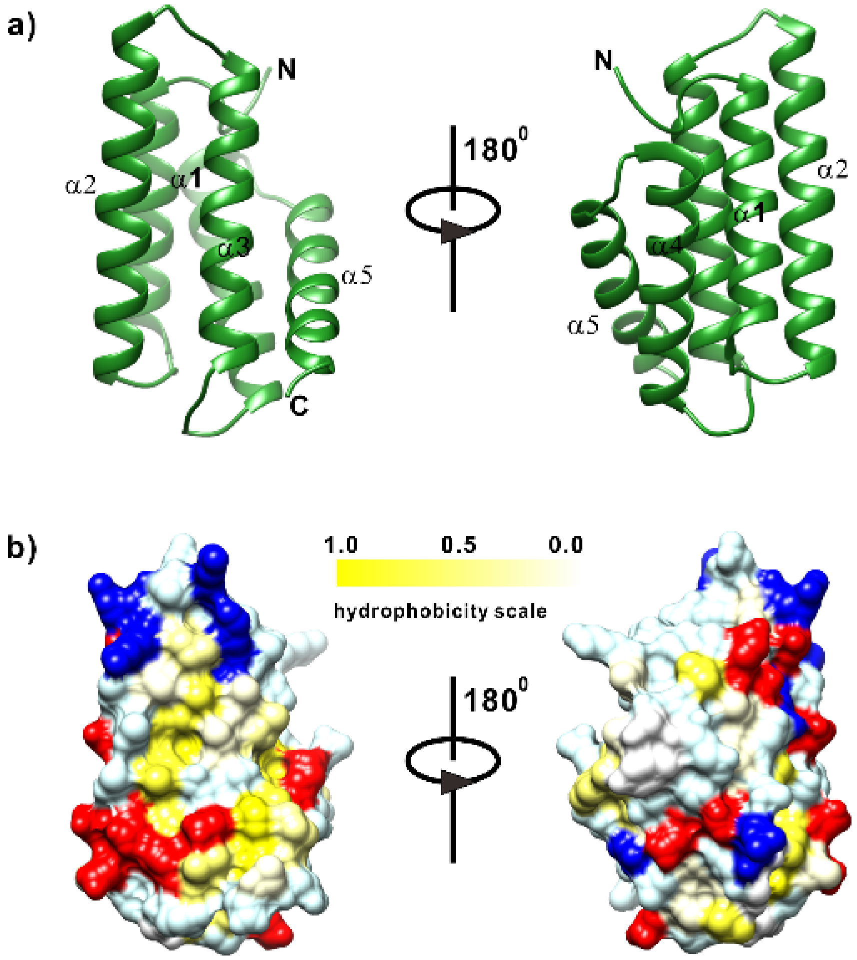

Figure S1: Monomeric structure of MaSpN is represented by (a) Ribbon diagram; (b) Hydrophobicity surface. Red and blue colors represent negatively and positively charged residues, respectively, in (b).

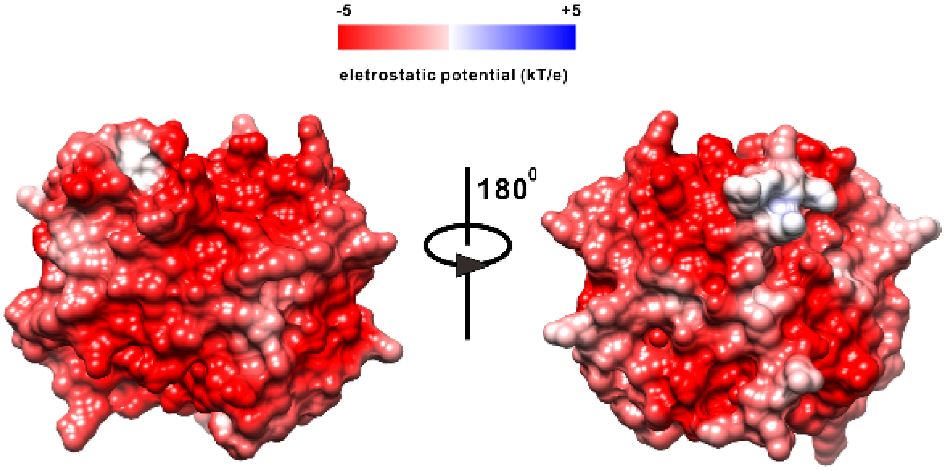

Figure S2. Electrostatic potential surface of MaSpN dimer.

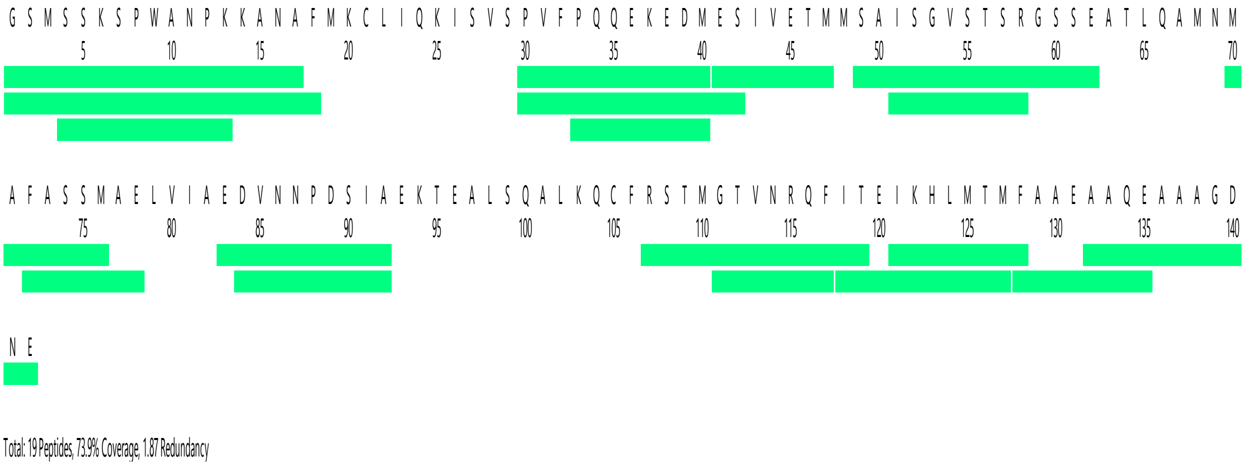

Figure S3. Amino acid sequence coverage map of AcSpN at pH 6.0 and 7.0 perturbations in HDXMS experiments. The detected peptides were indicated in green.

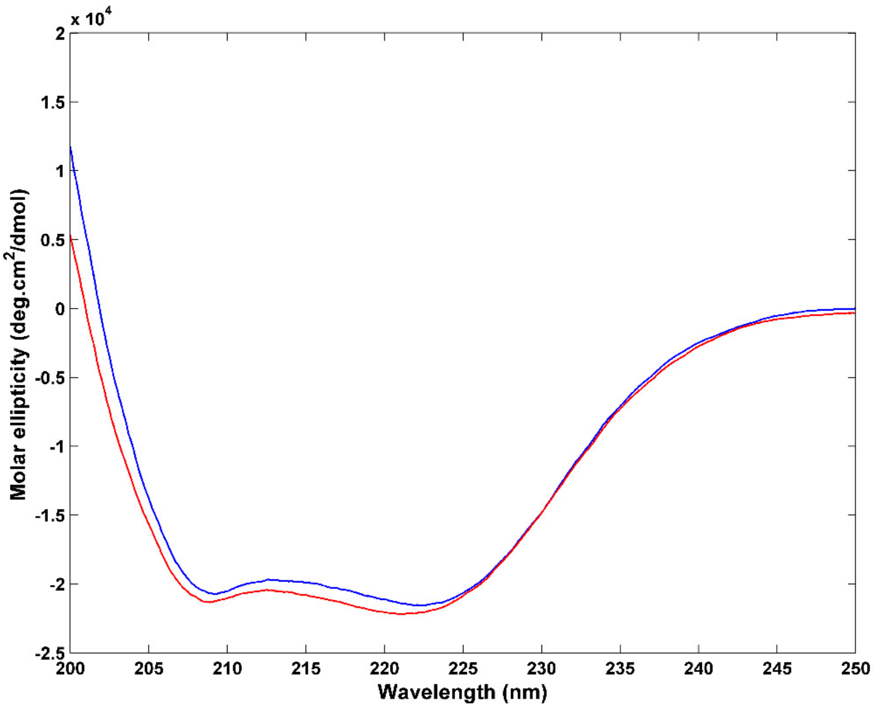

Figure S4. CD spectra of AcSpN at pH 6.0 (blue) and 7.0 (red) in the presence of 150 mM NaCl.

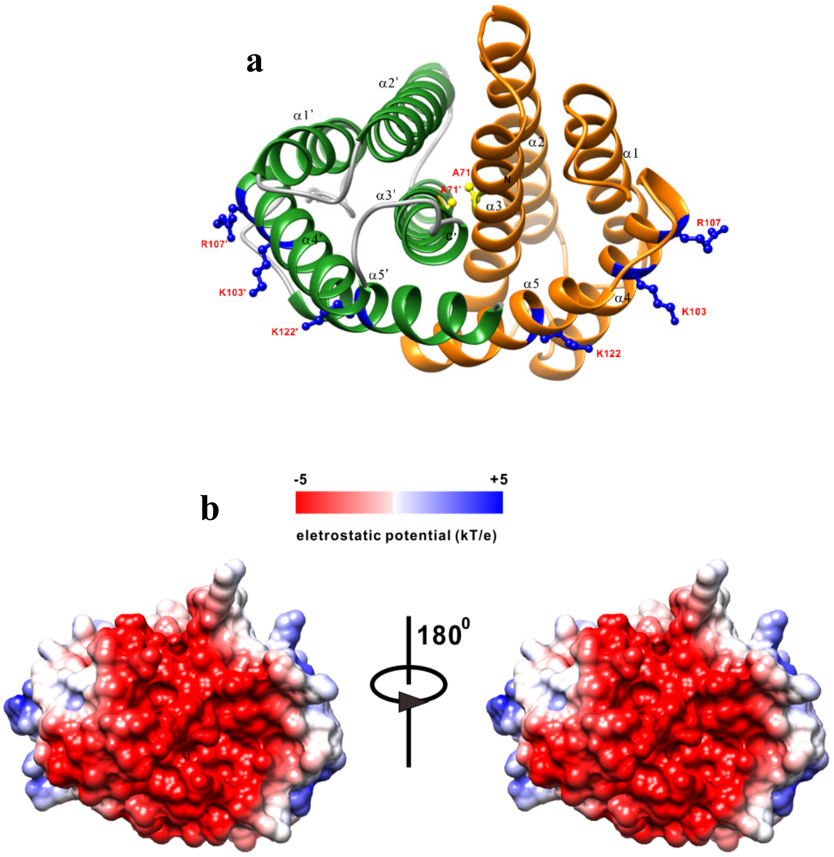

Figure S5. Dimeric structure of AcSpN obtained by assuming 41-47, 70-78 and 118-128 are located in the dimer interface. (a). Ribbon diagram. The positively charged residues (K103, R107 and K122) which were mutated into negatively charged residues are highlighted by blue sticks-and-balls. A71 and A71' are highlighted in yellow sticks-and-balls. (b). Electrostatic potential surface of the dimer.
